# Localized management of non-indigenous animal domesticates in Northwestern China during the Bronze Age

**DOI:** 10.1038/s41598-021-95233-x

**Published:** 2021-08-03

**Authors:** Petra Vaiglova, Rachel E. B. Reid, Emma Lightfoot, Suzanne E. Pilaar Birch, Hui Wang, Guoke Chen, Shuicheng Li, Martin Jones, Xinyi Liu

**Affiliations:** 1 Department of Anthropology, Washington University in St. Louis, 1 Brookings Dr., St. Louis, MO 63130 USA; 2 Department of Geosciences, Virginia Polytechnic Institute and State University, 926 West Campus Dr., Blacksburg, VA 24061 USA; 3 McDonald Institute for Archaeological Research, University of Cambridge, Downing St., Cambridge, CB2 3ER UK; 4 Department of Anthropology, Department of Geography, University of Georgia, 355 South Jackson Street, Athens, GA 30602 USA; 5 Department of Cultural Heritage and Museology, Fudan University, 220 Handan Rd., Shanghai, 200433 China; 6 Gansu Institute of Cultural Relics and Archaeology, 165 Heping Rd., Lanzhou, 730000 China; 7 Department of Archaeology, Sichuan University, 24 Yihuan Rd. South, Chengdu, 610065 China

**Keywords:** Environmental social sciences, Environmental economics

## Abstract

The movements of ancient crop and animal domesticates across prehistoric Eurasia are well-documented in the archaeological record. What is less well understood are the precise mechanisms that farmers and herders employed to incorporate newly introduced domesticates into their long-standing husbandry and culinary traditions. This paper presents stable isotope values (*δ*^13^C, *δ*^15^N) of humans, animals, and a small number of plants from the Hexi Corridor, a key region that facilitated the movement of ancient crops between Central and East Asia. The data show that the role of animal products in human diets was more significant than previously thought. In addition, the diets of domestic herbivores (sheep/goat, and cattle) suggest that these two groups of domesticates were managed in distinct ways in the two main ecozones of the Hexi Corridor: the drier Northwestern region and the wetter Southeastern region. Whereas sheep and goat diets are consistent with consumption of naturally available vegetation, cattle exhibit a higher input of C_4_ plants in places where these plants contributed little to the natural vegetation. This suggests that cattle consumed diets that were more influenced by human provisioning, and may therefore have been reared closer to the human settlements, than sheep and goats.

## Introduction

Between the 6th and the 2nd millennium BCE, crops and animals that were first domesticated on opposite ends of Eurasia were transported across long distances and adopted by communities in markedly different environments across the continent^1–9^. The exchange of crops native to Southwestern (SW) Asia (including free-threshing wheat, *T.* cf. *aestivum*, and barley, *H. vulgare*), with crops native to northern (N) China (including broomcorn millet, *P. miliaceum*, and foxtail millet*, S. italica*), enabled the creation of new agricultural systems that involved multi-seasonal cultivation of both indigenous and nonindigenous grains^10,11^. Similarly, animals that were domesticated in SW and Central Asia (which include sheep, goat, cattle, and horse) were brought into contact with a long-standing tradition of pig and dog rearing in ancient China, transforming animal-based subsistence and food production in this part of the world^12–16^.

Recent discussion has highlighted the importance of social context in culinary innovation and technological connectivity^1,17–19^. In the case of cereals, the reaction of an existing social and culinary system to novel crops appears to have been a key driver of their adoption and translocation in this region^20,21^. However, questions remain concerning the manner in which animal products were integrated into local management traditions and the degree to which this was affected by varying local microclimates.

This study aims to assess animal husbandry strategies and the role of these animals in human diets at nine archaeological sites from the Hexi Corridor in NW China, a region that is key to understanding trans-Eurasian movements of cereals and animals. The study integrates new (*n* = 210: 5 humans, 199 animals, 6 plants) and previously published (189 humans^19^, 48 humans^22^, 3 plants^23^; 167 animals published as summary statistics^23^) stable carbon (*δ*^13^C) and nitrogen (*δ*^15^N) isotope values from sites situated in the drier Northwestern (NW) and the wetter Southeastern (SE) regions of the Hexi Corridor. Six archaeological sites represent communities living in the NW region: Xihetan (XIH), Huoshaogou (HUO), Ganguya (GAN), Sanbadongzi (SAN), Wuba (WUB), and Mozuizi (MOZ). Three sites represent communities in the SE region: Mogou Cemetery (MOG-C), Mogou Settlement (MOG-S), and Zhanqi (ZHQ). The data enable an assessment of the varied management strategies that communities in distinct climatic zones within the Hexi Corridor developed for integrating the non-indigenous domesticates from Southwestern Asia into their agrarian spheres. The discussion widens our understanding of how these agrarian innovations fueled the overarching process of prehistoric Old World food globalisation.

### Geographic context: the Hexi Corridor

**Figure 1:**
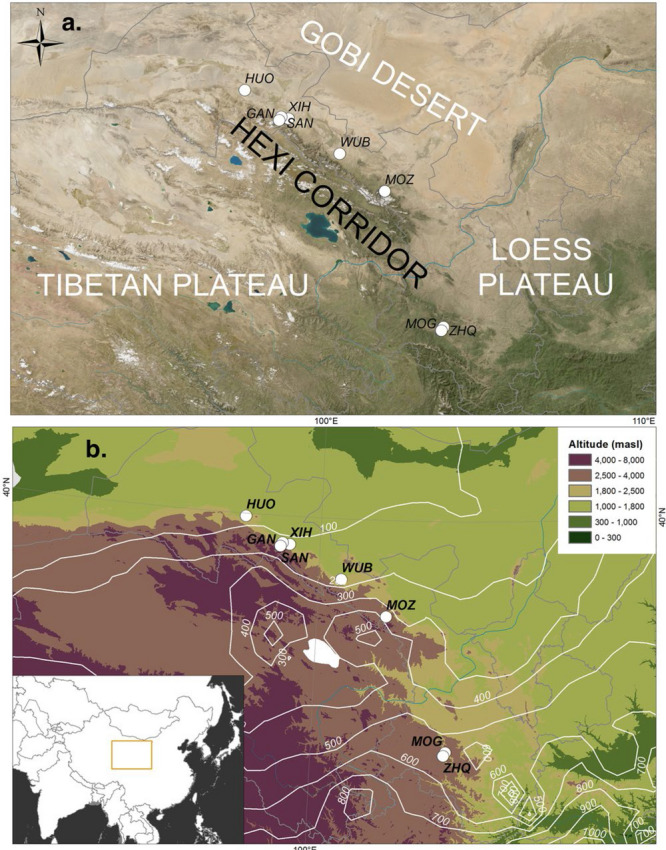
Maps of the study region. (**a**) Topography of the region. (**b**) Precipitation zones (indicated with white contours). *HUO* Huoshaogou, *SAN* Sanbadongzhi, *GAN* Ganguya, *XIH* Xihetan, *WUB* Wuba, *MOZ* Mozuizi, *MOG* Mogou, *ZHQ* Zhanqi. Maps generated using ArcGIS ArcMap 10.2 (https://www.esri.com/about/newsroom/arcwatch/the-best-of-arcgis-10-2/) and public domain data obtained from NASA Blue Marble (https://visibleearth.nasa.gov/collection/1484/blue-marble).

A key driver of ecological diversity across the vast landscape of China is the system of seasonal monsoons. The East Asian Summer Monsoon brings water from the Pacific Ocean into Eastern and Southern China and increases water availability in the arid regions of the Continental Interior situated north of the Tibetan Plateau^24^. One of these areas is Gansu Province, which lies within and just beyond the reach of the summer monsoon. It hosts a diverse topography, with a series of mountain ranges and lowland ecosystems between the Tibetan Plateau to the south and the Mongolian Gobi Desert to the north: the Hexi Corridor (Fig. 1). The SE region of the Hexi Corridor hosts a wetter climate, while the NW region, which borders the desert, is characterised by more arid terrains.

During the Bronze Age (2000–1000 BCE), the Hexi Corridor provided an important pathway connecting Central China with the eastern Eurasian Steppe. It facilitated the exchange of crops and domestic animals between agro-pastoralist communities in Central Asia and farming communities in Central/East China^22,23,25,26^. Archaeological material from this crossroads provides evidence for the spread of not only plant and animal domesticates but also material culture including chariot technology, metallurgy, burial traditions, and mudbrick (moving eastwards)^18,27–32^ and painted pottery (moving westwards)^27,33^.

Paleodietary reconstructions using stable isotope analyses suggest that human populations inhabiting the Hexi Corridor experienced a dietary shift in the early 2nd millennium BCE. Prior to 1900–1800 cal. BCE, local human diets largely consisted of eastern-originating millet products (and products from animals that subsisted on millet/other C_4_ plants). After this date, human diets were dominated by wheat and barley, as evidenced by a shift from stable carbon isotope values characteristic of C_4_ crop consumption to those characteristic of mixed C_3_/C_4_ crop consumption^19,22,34^.

Several authors^22,35,36^ explain this dietary transition as a result of the 4.2 ka BP global aridification event^37^, arguing that the region became too cool and dry for the cultivation of millet. However, this proposition does not explain why millet continued to be the staple crop in the Loess Plateau and the Central Plains China—which were also influenced by the 4.2 ka BP event—for another 2000 years after it ceased to be a staple in the Hexi Corridor. Climate changes alone can also not explain why the ecologically hardy broomcorn millet and foxtail millet spread across Eurasia to Eastern Europe immediately following the 4.2 ka BP event^38–40^. It is proposed here that the local cuisines played a role in the adoption/dismissal of these crops in Eastern China. Because the introduction of wheat and barley into the Hexi Corridor was likely accompanied by the flour-based grinding and baking cuisines from the West, these crops were readily adapted here by the local communities. Their lower adaptability to the boiling and steaming tradition characterizing the culinary system to the East, however, meant that they were initially rejected by communities in the Loess Plateau^21,23^.

At the same time that the staple grains were shifting, communities in the Hexi Corridor were integrating SW Asian sheep, goat, and cattle into their local husbandry systems. To better understand the mechanism of this change, this study aims to assess animal husbandry strategies in the Hexi Corridor during the Bronze Age. Were the new species managed in similar ways as locally domesticated dogs and pigs? Did they graze near or far from human settlements? What was the role of these domestic herbivores and omnivores in the diets of the people living there?

## Results

### Pigs, dogs, cattle, sheep/goat

**Figure 2:**
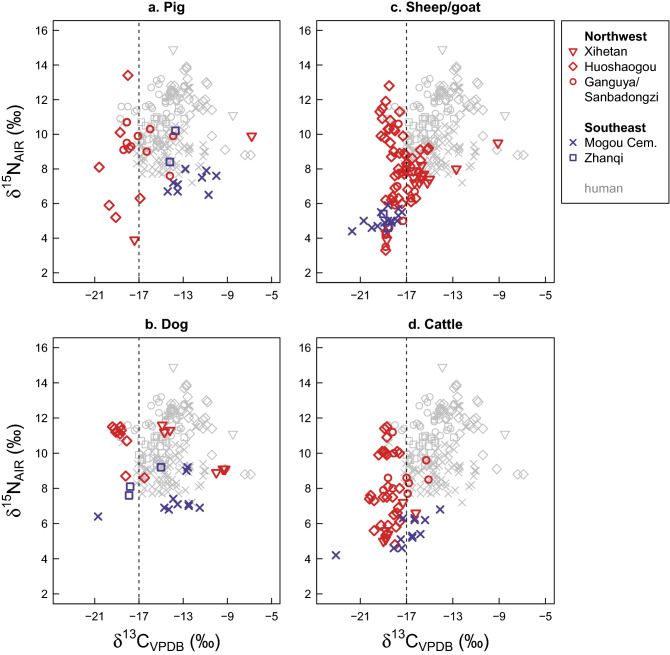
Stable isotope (carbon, nitrogen) values of humans and major animal species from sites post-dating 1900 cal BCE. Bivariate plots showing *δ*^13^C and *δ*^15^N values of (**a**) pig, (**b**) dog, (**c**) sheep/goat, and (**d**) cattle. The sites are color-coded according to their location in the Southeastern or Northwestern regions of the Hexi Corridor. The human data are presented for comparison using grey symbols. The dashed line at − 17‰ indicates the approximate boundary between a predominantly C_3_-based diet (to the left of the line) and a mixed C_3_/C_4_ diet (to the right of the line).

The data show that in the 2nd millennium BCE, pigs from the Northwestern (XIH, HUO, GAN/SAN, *n* = 19) and the Southeastern (MOG-C, ZHQ, *n* = 12) regions had distinct diets (Fig. 2a). In the Southeast, pigs exhibit *δ*^13^C values that range from − 14.4 to − 10.0‰. Within each site, mean pig *δ*^13^C values are less negative than mean human *δ*^13^C values (MOG-C: pigs − 12.5 ± 1.5‰, humans − 14.3 ± 1.7‰; ZHQ: pigs − 14.0 ± 0.4‰, humans − 15.3 ± 1.0‰). In the Northwest, most pig *δ*^13^C values cluster between − 20.6 and − 13.9‰, with one pig from XIH (JX52) exhibiting a value of − 6.8‰. Within each site, mean pig *δ*^13^C values are more negative than those of the humans (HUO: pigs − 18.7 ± 1.2‰, humans − 12.2 ± 1.8‰; GAN/SAN: pigs − 16.8 ± 1.7‰, humans − 15.3 ± 1.5‰). These patterns indicate that pig diets in the Southeastern region of the Hexi Corridor included higher amounts of C_4_ crop products and by-products than pig diets in the Northwest.

Unlike the pigs, the data do not indicate a pronounced Northwest–Southeast (NW–SE) division in the *δ*^13^C values of dogs (NW, *n* = 17: mean − 15.7 ± 3.9‰, ranging 10.2‰ from − 19.4 to − 9.2‰; SE, *n* = 13: mean − 14.6 ± 2.7‰, ranging 9.2‰ from − 20.7 to − 11.5‰) (Fig. 2b). At all sites except Mogou Cemetery, mean dog *δ*^13^C values are lower than those of the local humans (HUO: dogs − 18.6 ± 0.8‰, humans − 12.2 ± 1.8‰; XIH: dogs − 11.7 ± 2.8‰, humans − 11.2 ± 3.8‰; ZHQ: dogs − 16.9 ± 1.6‰, humans − 15.3 ± 1.0‰; MOG-C, dogs − 13.9 ± 2.9‰, humans − 14.3 ± 1.7‰).

Cattle and sheep/goat exhibit opposite patterns in the two regions (Fig. 2c,d). At the Northwestern sites, sheep/goat exhibit both pure C_3_ diets and mixed C_3_/C_4_ diets, with *δ*^13^C values of most samples (*n* = 68) falling between − 19.3‰ and − 15.0‰ and two samples from XIH exhibiting values of − 12.7‰ (JX205) and − 9.1‰ (JX204) (mean of all NW sheep/goat − 17.2 ± 1.6‰). Most of the cattle in this region (39/45) exhibit purely C_3_ diets with *δ*^13^C values between − 20.2‰ and − 17.0‰. Six individuals (5 from GAN/SAN, 1 from XIH) exhibit values from − 17.0‰ to − 15.1‰ (mean of all NW cattle − 18.3 ± 1.1‰). At the Southeastern sites, the trends are reversed. Sheep/goat exhibit pure C_3_ diets with *δ*^13^C values between − 21.7‰ and − 17.4‰ (mean: − 18.9 ± 1.1‰, *n* = 18). Cattle (only from MOG-C, *n* = 12) exhibit both pure C_3_ and mixed C_3_/C_4_ diets with *δ*^13^C values between − 23.1‰ and − 14.1‰ (mean: − 17.0 ± 2.2‰).

### Humans

**Figure 3:**
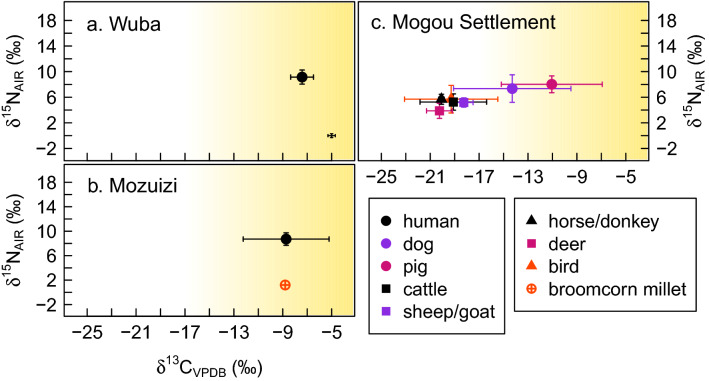
Stable isotope (carbon, nitrogen) results from sites pre-dating 1900 cal BCE. Bivariate plots of mean *δ*^13^C and *δ*^15^N values of human, plant, and animal samples from (**a**) Wuba, (**b**) Mozuizi and (**c**) Mogou Settlement. Variability is shown as standard deviation, 1 σ. The shading indicates increasing input of C_4_ vegetation in consumer tissues, with the cutoff set to − 17‰^41^. Measurement error shown in the bottom-right corner of (**a**). See Table 2 for a breakdown of sample numbers.

**Figure 4:**
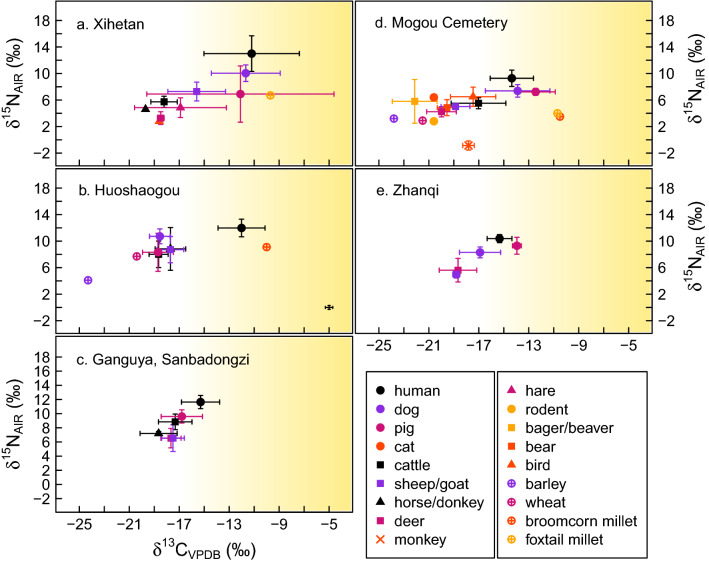
Stable isotope (carbon, nitrogen) results from sites post-dating 1900 cal BCE. Bivariate plots of mean *δ*^13^C and *δ*^15^N values of human, plant, and animal samples from (**a**) Xihetan, (**b**) Huoshaogou, (**c**) Ganguya and Sanbadongzi, (**d**) Mogou Cemetery, and (**e**) Zhanqi. Variability is shown as standard deviation, 1 σ. The shading indicates increasing input of C_4_ vegetation in consumers’ diets, with the cutoff set to − 17‰^41^. Measurement error shown in the bottom-right corner of (**b**). See Table 2 for a breakdown of sample numbers.

As published previously^19,21^, humans pre-dating the 1900 cal BCE transition (WUB and MOZ) exhibit *δ*^13^C values that are strongly influenced by C_4_ plant inputs (Fig. 3). Humans post-dating this transition (HUO, GAN/SAN, ZHQ) exhibit primarily mixed C_3_/C_4_ diets, with a small number of individuals in all groups (except for Huoshaogou) exhibiting C_3_-dominated diets (Fig. 4). Table 1 shows the summary statistics for each site and Supplementary Figs. S1 and S2 present the bivariate plots with all data.

**Table 1:** Summary statistics of all *δ*^15^N and *δ*^13^C values discussed in this study.

	*n* =	Mean *δ*^15^N	*δ*^15^N SD (1σ)	Mean *δ*^13^C	*δ*^13^C SD (1σ)
**Wuba**					
Human	55	9.1	1.1	− 7.4	0.9
**Mozuizi**					
Human	17	8.7	1.0	− 8.7	3.5
Broomcorn millet	1	1.2	–	− 8.8	–
**Mogou settlement**					
Sheep/goat	10	5.2	0.7	− 18.3	0.8
Cattle	10	5.3	1.3	− 19.1	2.7
Pig	9	8.0	1.3	− 11.1	4.1
Dog	10	7.4	2.1	− 14.3	4.8
Deer	31	3.9	1.2	− 20.2	1.1
Horse/donkey	6	5.7	0.8	− 20.1	0.2
Bird	7	5.7	2.2	− 19.3	3.8
**Xihetan**					
Human	2	13.0	2.7	− 11.2	3.8
Sheep/goat	15	7.3	1.4	− 15.6	2.3
Cattle	7	5.7	0.8	− 18.2	1.1
Pig	2	6.9	4.2	− 12.1	7.5
Dog	7	10.0	1.2	− 11.7	2.8
Deer	5	3.3	1.0	− 18.5	0.3
Horse/donkey	1	4.6	–	− 19.7	–
Hare	2	4.9	1.5	− 16.9	3.7
Bird	1	2.8	–	− 18.6	–
Foxtail millet	1	6.7	–	− 9.7	–
**Huoshaogou**					
Human	30	12.0	1.3	− 12.0	1.9
Sheep/goat	41	8.7	2.0	− 17.7	1.1
Cattle	28	8.0	2.0	− 18.7	0.8
Pig	7	8.3	2.9	− 18.7	1.2
Dog	10	10.7	1.1	− 18.6	0.8
Horse/donkey	4	8.8	3.2	− 17.7	1.2
Wheat	1	7.7	–	− 20.4	–
Barley	1	4.1	–	− 24.3	–
Broomcorn millet	1	9.1	–	− 10.0	–
**Ganguya, Sanbadongzi**					
Human	30	11.6	0.9	− 15.3	1.5
Sheep/goat	14	6.5	1.9	− 17.5	0.9
Cattle	10	8.8	1.1	− 17.3	1.3
Pig	10	9.6	0.9	− 16.8	1.7
Deer	8	6.5	1.4	− 17.7	0.8
Horse/donkey	2	7.2	0.3	− 18.7	1.5
**Mogou Cemetery**					
Human	85	9.3	1.2	− 14.4	1.7
Sheep/goat	16	5.0	0.5	− 18.9	1.2
Cattle	12	5.5	0.8	− 17.0	2.2
Pig	10	7.2	0.5	− 12.5	1.6
Dog	10	7.4	0.9	− 13.9	2.6
Deer	18	4.2	0.8	− 20.0	1.2
Badger/beaver	3	5.8	3.3	− 22.1	1.8
Bear	2	4.9	1.2	− 19.6	0.8
Bird	7	6.5	1.4	− 17.5	1.8
Cat	1	6.4	–	− 20.6	–
Monkey	16	-0.9	0.7	− 17.8	0.5
Rodent	1	2.8	–	− 20.6	–
Wheat	1	2.9	–	− 21.5	–
Barley	1	3.2	–	− 23.8	–
Broomcorn millet	1	3.5	–	− 10.5	–
Foxtail millet	1	4.0	–	− 10.7	–
**Zhanqi**					
Human	23	10.4	0.6	− 15.3	1.0
Sheep/goat	2	5.0	0.6	− 18.8	0.3
Pig	2	9.3	1.3	− 14.0	0.4
Dog	3	8.3	0.8	− 16.9	1.6
Deer	6	5.6	1.8	− 18.7	1.5

At Huoshaogou, the human *δ*^13^C values are completely separate from the animal values (humans, *n* = 30: − 12.2 ± 1.8‰, ranging 7.2‰ from − 14.1‰ to − 6.9‰; animals, *n* = 90: − 18.2 ± 1.1‰, ranging 5.5‰ from − 20.6‰ to − 15.1‰). At Ganguya and Sanbadongzi, the *δ*^13^C values of humans and animals show a near-total overlap (humans, *n* = 30: − 15.3 ± 1.5‰, ranging 5.8‰ from − 18.7‰ to − 12.9‰; animals, *n* = 44: − 17.4 ± 1.2‰, ranging 5.8‰ from − 19.7‰ to − 13.9‰).

### Plants

Bulk crop grains were measured in small numbers from both Northwestern and Southeastern regions (Figs. 3 and 4). Broomcorn millet (*n* = 3, from HUO, GAN/SAN, and MOG-C) exhibits a narrow range of *δ*^13^C values (− 10.5‰ to − 8.8‰) and a wide range of *δ*^15^N values (+ 1.2‰ at HUO to + 9.1‰ at GAN/SAN; with a sample from MOG-C at + 3.5‰). Foxtail millet (*n* = 2, from MOC-C and HUO) exhibits *δ*^13^C values of − 10.7‰ and − 9.7‰ and *δ*^15^N values of + 4.0‰ and + 6.7‰, respectively. Barley (n = 2, from MOG-C and GAN/SAN) exhibits lower *δ*^13^C values than wheat (*n* = 2, from GAN/SAN and MOG-C) (− 24.3‰ and − 23.8‰ for barley and − 21.5‰ and − 0.4‰ for wheat). The wheat *δ*^15^N values bracket those of the barley (+ 3.2‰ and + 4.1‰ for barley and + 2.9 and + 7.7‰ for wheat). With converted Δ^13^C values (cf.^42^) of + 17.6‰ and + 18.1‰ and situated above the ‘poorly watered band’, the barley samples appear to have been grown in water conditions that were not limiting to growth. The wheat, on the other hand, appears to have been grown in suboptimal watering conditions, with Δ^13^C values of + 14.1‰ and + 15.2‰.

### δ^15^N value offset between humans and animals

**Figure 5:**
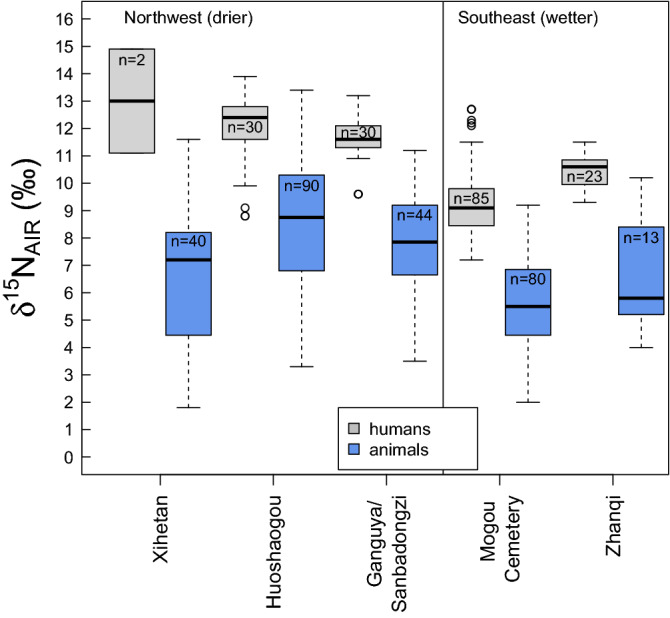
Stable nitrogen isotope values of humans and animals from sites post-dating 1900 cal BCE. Boxplots indicating the minimum, first quartile, median, third quartile, maximum and outlying (open circles) *δ*^15^N values of humans and animals from each site. Monkeys from Mogou Cemetery are not included.

**Figure 6:**
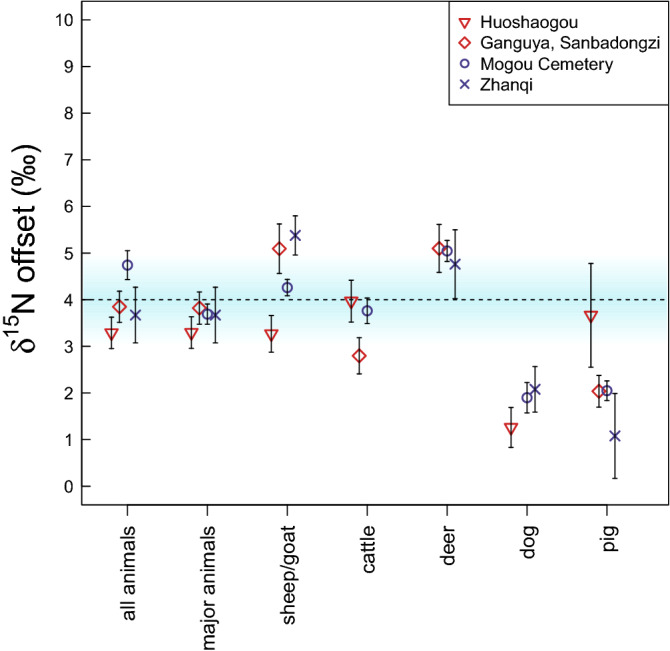
Stable nitrogen isotope offsets between humans and animals from sites post-dating 1900 cal BCE. Univariate plots showing the difference between the means of humans and the means of: all animals (including monkeys from Mogou Cemetery); major animals (pig, dog, sheep/goat, cattle, deer) combined; and each of the major animals individually. The error bars indicate standard error, 1 σ. The dashed line and shading indicate the average diet–tissue offset of 3–5‰^44–46^. Xihetan is not included because the site only includes 2 human samples, so the offsets would not be meaningful.

Human and animal *δ*^15^N values from the period post-dating 1900 cal BCE differ between regions (humans: NW + 11.9 ± 1.1‰, SE + 9.6 ± 1.2‰; animals: NW + 8.0 ± 2.3‰; SE + 4.8 ± 2.8‰). The differences are statistically significant at 99% confidence (two-tailed non-paired equal variance t-test between NW humans & SE humans: *t* = 12.63, *df* = 232, *p* < 0.01; two-tailed non-paired equal variance t-test between NW animals & SE animals: *t* = 15.89, *df* = 211,* p* < 0.01) (Fig. 5). This suggests that the nitrogen isotope baseline is elevated in the Northwestern Hexi Corridor due to higher temperatures and lower mean annual rainfall^43^. Apart from Xihetan, where human *n* = 2, the *δ*^15^N offset between humans and animals at each site is within the 3–5‰ diet–tissue enrichment factor (HUO: 3.5‰; GAN/SAN: 3.8‰; ZHQ: 3.7‰; MOG-C: 4.7‰ with monkeys included, 3.8‰ with monkeys removed). The offsets between humans and most abundant animal species (pig, dog, cattle, sheep/goat, deer) are identical to the offsets between humans and all animals (HUO: 3.5‰; GAN/SAN: 3.8‰; ZHQ: 3.6‰; MOG-C: 3.7‰) (Fig. 6). *δ*^15^N offsets between humans and major animal species (sheep/goat, cattle, deer, dog, pig) are not consistent across regions (Fig. 6). The offsets with sheep/goat, cattle and deer are close to the diet–tissue enrichment of 3–5‰, while the offsets with dogs and pigs are lower (except at Huoshaogou).

### Monkeys

The macaque monkeys from Mogou Cemetery exhibit negative *δ*^15^N values (− 0.9 ± 0.7‰, ranging 2.3‰ from − 2.0‰ to + 0.3‰, *n* = 16). Although monkeys have been found to exhibit low *δ*^15^N values as a result of eating both leguminous and non-leguminous plants from moist forest floors^47^, no negative *δ*^15^N values from monkeys have been reported in the literature to date, and the mechanism for incorporation of ^15^N-depleted nitrogen remains to be explained.

## Discussion

### Role of animal foods in human diets in the Hexi Corridor

The difference in mean *δ*^15^N values between the humans and animals in this study lie within the generally accepted 3–5‰ interval for trophic enrichment^48^. This suggests that, as far as protein intake was concerned, animal products had more than a minimal role in human diets. This disagrees with the suggestion (from an earlier study based on limited sample sizes) that animal products played a minor role in human diets in the Northwestern part of the Hexi Corridor^34^. The results presented here suggest that animal products were consumed in sufficient amounts to drive protein intake, but not so much as to dominate both protein and energy intake. This stands in contrast to human diets in Central China, where it has been argued that historically, human subsistence was primarily based on grain consumption^49,50^.

To assess whether the protein component of human diets was driven by a particular domestic animal species, individual offsets in *δ*^15^N values were calculated between humans and the major animal taxa (sheep/goat, cattle, deer, dog, and pig). The data suggest that the offsets between humans and the omnivores (dogs and pigs) are lower than the 3–5‰ trophic enrichment interval, except at Huoshaogou. This indicates that animal protein intake was not limited to omnivore meat, but must have included additional sources.

The offsets between humans and herbivores (sheep/goat, cattle, deer) lie closer to the trophic enrichment interval, ranging from 2.5–6‰. However, the lack of any systematic patterns between the sites suggests that meat and dairy intake consisted of varied combinations of species at the different locations. Animal products were obtained either primarily from animals whose offsets lie close to 4‰ (i.e., sheep, goat and cattle), with smaller inputs from the remaining species (deer, dog, and pig), or in equal amounts from animals that lie above and below the 4‰ offset (for example, deer and pig at GAN/SAN and MOG-C). No two sites exhibit the same combination of *δ*^15^N offsets, making it unlikely that the communities in different locations followed the same dietary patterns. Instead, diets across the Hexi Corridor were heterogeneous: some communities and individuals may have chosen to consume diets dominated by sheep, goat, and cattle, while others preferred more deer, pig, and dog.

### New insight into Bronze Age animal husbandry in the Hexi Corridor

The feeding patterns of dogs, pigs, and domestic herbivores at the study sites, as inferred from stable isotope analyses, provide insight into how prehistoric populations in the Hexi Corridor integrated animals that had been domesticated in the region for millennia with those that had been recently introduced into their agricultural and social spheres.

Prior to the introduction of domesticates from Southwestern Asia in the 2nd millennium BCE, communities in Northern China practiced a subsistence economy based on millet cultivation and pig husbandry. These two spheres of the Neolithic economy were tightly integrated, as evidenced by the pigs’ consumption of millet products and byproducts dating back to c. 5700 BCE^14,51^. Liu and Jones^52^ argue that pigs were kept in social enclosures, which restricted their movement and reoriented their dependence on natural vegetation towards agricultural fodder and the leftovers of human food. Accordingly, the similarity of pig and human diets is evident both in early Neolithic contexts at the sites of Dadiwan and Xinglonggou^14,51^, and at Middle and Late Neolithic sites across North China^16,52–54^.

In this study, the pig *δ*^13^C values lie within ~2‰ of the *δ*^13^C values of the humans, except at Huoshaogou, where pig *δ*^13^C values indicate a notably lower consumption of C_4_ plants. This suggests that even within pig rearing—the agricultural sphere that had a long-standing tradition in the region—people across the Hexi Corridor made choices that broke with tradition and adopted a management strategy that enabled them to conceive of human and pig foods as separate.

With the arrival of the Southwestern Asian domesticates, farmers in the Hexi Corridor had a choice to either integrate the animals into the existing stall-feeding/millet-foddering system used for pig raising at Ganguya, Sanbadongzhi, Mogou and Zhanqi, or employ a local pastoral strategy, such as the one used for managing pigs at Huoshaogou. As a result of this choice, localised distinctions in animal herding strategies arose in different parts of the region, reflecting varying degrees to which the non-locally domesticated animals were integrated into the long-standing pig–millet economy.

Sheep and goats in both the Northwestern and the Southeastern regions exhibit diets primarily composed of naturally available plants, suggesting that they were managed within the local grazing landscape. In the wetter Southeastern Hexi Corridor, sheep/goat exhibit pure C_3_ diets, while in the drier NW region, their carbon isotope values indicate consumption of both C_3_ and C_4_ vegetation. This is consistent with the natural spread of vegetation in these regions, with higher amounts of C_4_ plants occurring in the NW Hexi Corridor^55^, owing to C_4_ plants’ higher proclivity to aridity and solar radiation^56,57^. Therefore, in the NW, sheep/goat diets reflect either occasional/seasonal consumption of C_4_ vegetation or consumption of water-stressed C_3_ plants with less negative *δ*^13^C values^42,58,59^. These values are consistent with previously published data for herbivores from hot/arid environments, whose elevated *δ*^13^C values have been explained by the composition of their diet rather than by a physiological response to hotter environments^60^.

The diets of cattle exhibit the opposite patterns to the sheep/goat, with individuals from the drier NW Hexi Corridor exhibiting pure C_3_ diets and those raised in the wetter SE region exhibiting *δ*^13^C values up to − 14‰, indicative of C_4_ plant consumption. At the Northwestern sites of Huoshaogou, Ganguya and Sanbadongzhi, cattle subsisted on more restricted diets compared to the sheep and goats, probably because cattle lacked access to the arid-adapted C_4_ or water-stressed C_3_ plants.

In the Southeastern region, cattle were only available from Mogou, which is situated at an altitude of 2200 masl (meters above sea level). Distribution of C_4_ vegetation declines at high elevations^61^, with C_3_-dominated landscapes occurring above ~1500–3000 masl, depending on the continent. This suggests that any C_4_ signatures in cattle raised at Mogou are unlikely to be the result of grazing on naturally occurring C_4_ plants in the immediate surroundings of the settlement. Instead, they may have either been fed cultivated C_4_ crop products/byproducts or moved seasonally to lower altitudes with native C_4_ grasses. Ongoing research of sequential tooth enamel carbonate isotope analysis will help clarify the seasonal dietary and mobility patterns of these animals.

A parallel can be drawn between the results of the current study and inferences of similar practices from the southern Levant. At the Chalcolithic site of Marj Rabba^62^, in Jordan, wider variation in cattle *δ*^13^C values compared to sheep/goat *δ*^13^C values is interpreted to indicate that two foddering/pasturing strategies were employed for cattle: one which involved local grazing and another which may have involved winter foddering on C_3_/C_4_ fodder and/or mobility outside of the local region. Similar isotopic distinctions between sheep/goat and cattle can be seen in Northern China^53,63^. At Xinzhai, stable isotope analysis of tooth enamel carbonate shows that, in contrast to sheep/goat diets, cattle diets included high amounts of seasonal fodder (likely C_4_ millet), which was interpreted to indicate that these animals were managed closer to the settlements than the sheep/goats^64^. The reverse pattern has been observed in the Dzhungar Mountains during the 3rd millennium BCE, where cattle exhibited predominantly C_3_ diets, while sheep and goats were seemingly foddered with C_4_ plants during the winter^65^.

These conclusions resonate with modern ethnographic examples from North China, where cattle have been observed to be more tethered to human settlements, rather than being allowed to roam on local or more distant pastures alongside sheep and goats^66^. These distinctions in herding practices are likely a result of either physio-behavioral differences between the two animals or varying assignment of culinary/cultural values. Sheep and goats tolerate rocky, frosty, and arid environments, whereas cattle require more water and are less resilient to extreme temperatures^66^. In Bronze Age China, cattle likely enjoyed high sacrificial value for ritual activities, as documented by early textual records^67^. The prestige and power associated with this status may have been a reason that cattle were kept closer to settlements rather than allowed to roam in more distant pastures with sheep and goats.

In summary, a contrast can be drawn between herding practices in the NW and SE regions of the Hexi Corridor. In the NW, sheep and goats may have been taken to graze on the fringes of the Gobi Desert and the foothills/hillsides of the Qilian Mountains, where they would have had access to C_4_ plants and water-stressed C_3_ plants. Cattle would have tolerated these landscapes less easily and would therefore have needed to graze closer to the oases or rivers where the settlements were located. This type of pastoral system is evidenced with the recent discovery of a large corral (over 200 m^2^) at Xihetan^68^. In the SE, on the other hand, Mogou is situated in a spatially constrained valley between the highlands and multiple lower-elevation catchment zones along the Tao River. In a location with limited grazing lands, allowing cattle to roam on land otherwise suitable for farming activities would have created significant socio-political tensions. A strategy that relied on seasonal pasturing of sheep and goats and stall-feeding of cattle would have provided farmers with an optimal solution representing continuity with the long-lasting Neolithic tradition of pig rearing^52^.

## Implications

The insight gained into animal management in Bronze Age Hexi Corridor has implications for:

1. the role of animal products in local human diets,
2. the heterogeneous nature of human diets, and
3. the relationship between newly introduced domestic animals and local rearing traditions.

The well-documented human dietary changes in the early 2nd millennium BCE in Northwestern China were partly driven by the consumption of newly introduced domestic herbivores. Previous discussions have primarily focused on the shifts in staple grain consumption from millet towards SW Asian cereal crops. This paper argues for increased role of animal products in human diets. While wheat and barley were gaining the status of staple crops, the consumption of sheep, goats, and cattle was also increasing.

In the broader regional context, the Hexi Corridor and the Loess Plateau present opposing dietary patterns in the 2nd millennium BCE. People in the Hexi Corridor adopted Western grains rapidly, while those in the Loess Plateau neglected them. Although the results of this study constitute only a local assessment, it appears that the wide range of subsistence activities employed in the Hexi Corridor contrasts with the unified millet-based agrarian practice widespread across the Loess Plateau. From this perspective, the regional differences can partially be explained by differences in subsistence economies: sedentary farming in the Loess Plateau versus multi-resource agro-pastoralism in the Hexi Corridor. This in turn challenges the traditional narrative of ‘modes’ of subsistence—hunting, foraging, pastoralism, and farming—progressing and developing in a linear evolutionary framework. The results from the Hexi Corridor show that people moved fairly readily between varying modes of subsistence and coexisted with neighboring populations that employed different modes. It has been demonstrated, ethnographically and archaeologically, that the same people may have practiced more than one subsistence mode in a single lifetime, reflecting the choices of people under certain social and environmental conditions rather than a proscribed stage of ‘economic development’^21,69,70^.

The third inference concerns the relationship between non-locally domesticated plants/animals and indigenous culinary and rearing traditions. The social context in which agricultural and culinary innovations occurred across prehistoric Eurasia has been heavily debated^19,71^. Emphases have been placed on the reaction of an existing social and culinary system to novel crops or the role of technology as a key driver of their adoption and translocation. In the context of Southeastern dispersal of metallurgical traditions, for example, Rawson^31^ suggested that when foreign innovations were adopted in ancient Central China, they were transformed within highly organized social and cultural systems, and this was particularly pronounced in the adoption of bronze casting technique within Eastern ritualistic contexts. In the case of eastward expansion of wheat, it has been demonstrated that this process likely exerted selection for phenotypic traits that were particularly suited to the eastern boiling and steaming tradition^23^. Both these ‘transformations’ initially occurred in an area that included the Southeastern Hexi Corridor. Our results suggest a similar process in the adoption of cattle: in locations with limited grazing lands suitable for pasturing of cattle, people adapted the local pig rearing economy towards cattle stall-feeding.

## Materials and methods

Archaeological human (*n* = 194), animal (*n* = 366), and plant (*n* = 9) samples were obtained from nine sites in the Hexi Corridor of Gansu Province and measured for *δ*^13^C and *δ*^15^N values (Fig. 1, Table 2). Most of the human data (*n* = 189) has been published previously^19^. The summary statistics of a portion of the animal dataset (*n* = 167) and values of three plant samples (from Huoshaogou) were used in previous human diet modelling^23^. This is the first time that all the data is reported in full. Forty-eight human samples from Mogou Cemetery measured by Ma et al.^22^ are included here for comparison, bringing the total of human samples to 242.

**Table 2:** Overview of all human, animal and plant samples from each study site.

Site	Culture	Culture date	Time group^a^	Human (*n* =)	Animal (*n* =)	Plant (*n* =)	Published in Liu et al.^19^	Summary statistics in Liu et al.^23^
Wuba (WUB)	Banshan, Machang, Qijia, transitional	4450–3600 cal. BP	Mostly pre-1900 cal BCE, some post-1900 cal BCE dates	55	–	–	55 humans	
Xihetan (XIH)	Qijia, Siba	4000–3600 cal. BP, 3700–3300 cal. BP	Post-1900 cal BCE	2	40	1		33 animals
Mozuizi (MOZ)	Machang, historical	4200–4000 cal. BP	Mostly pre-1900 cal BCE, some post-1900 cal BCE dates	17	–	1	14 humans (excl. WM102, WM103, WM104)	
Huoshaogou (HUO)	Siba, Shanma	3700–3300 cal. BP, 2900–2100 cal. BP	Mostly post-1900 cal BCE, some pre-1900 cal BCE	30	90	3	30 humans	90 animals
Ganguya (GAN)	Siba	3700–3300 cal. BP	Post-1900 cal BCE	30	–	–	30 humans	
Sanbadongzi (SAN)	Siba	3700–3300 cal. BP	Post-1900 cal BCE	–	44	–		44 animals
Mogou Settlement (MOG-S)	Qijia, Siwa	4000–3600 cal. BP, 3400–2500 cal. BP	Pre-1900 cal BCE	–	83	–		
Mogou Cemetery (MOG-C)	Qijia, Siwa	4000–3600 cal. BP, 3400–2500 cal. BP	Post-1900 cal BCE	85^b^	96	4	37 humans (codes starting with CM)	
Zhanqi (ZHQ)	Siwa	3400–2500 cal. BP	Post-1900 cal BCE	23	13		23 humans (codes MZ001-024)	

^a^From Ma et al.^22^ and Yang et al.^16^.

^b^48 of the human samples were published by Ma et al. (codes starting with M86 and MGM).

Six sites (Wuba, Mozuizi, Xihetan, Ganguya, Sanbadongzi, and Huoshaogou) are located in the drier Northwestern part of the region, while three (Mogou Settlement, Mogou Cemetery, and Zhanqi) are located in the wetter Southeastern region. Due to their geographic and temporal proximity, Ganguya and Sanbadongzi are grouped together and treated as one analytical unit. All sites have been assigned to time periods of either ‘pre-1900 cal BCE’ or ‘post-1900 cal BCE’ using established cultural chronologies and radiocarbon dates^19^.

Animal samples were identified to the lowest taxonomic level possible based on qualitative morphological traits. The current study was aimed at identifying samples suitable for isotopic analysis and did not provide a full overview of the composition of the zooarchaeological assemblage; more comprehensive analysis is still ongoing and will provide complementary information. Domestic taxa include sheep (*Ovis aries*), domestic goat (*Capra hircus*), domestic cattle (*Bos taurus*), domestic dog (*Canis familiaris*), domestic pig (*Sus scrofa*), and equids (*Equus* sp., horse/donkey). Wild animals included deer (taxon unspecified), monkeys (macaques, *Macaca* sp.), cat (*Felis* sp.), hare (taxon unspecified), rodent (taxon unspecified), badger (*Meles* sp.) and/or beaver (*Castor fiber*), bear (*Ursus* sp.), and multiple birds (taxon unspecified).

Bone collagen was isolated using a modified Longin^72^ protocol^73^. Only samples with acceptable collagen C/N ratios (2.9–3.6^74^) are reported. Plant samples represent homogenous mixtures of 2–8 grains (for barley, *Hordeum vulgare*, and wheat, *Triticum aestivum*) and 15–22 grains (for broomcorn millet, *Panicum miliaceum,* and foxtail millet*, Setaria italica*) per archaeological context (see Supplementary Table S1). The samples represent the best-preserved grains at the site. The plant isotopic values were corrected for a charring offset of + 0.1‰ in *δ*^13^C values and + 0.3‰ in *δ*^15^N values following Nitsch et al.^75^. The barley and wheat *δ*^13^C values were converted to Δ^13^C values to enable comparison of the carbon isotope discrimination of the samples to the ‘watering thresholds’ established by Wallace et al.^59^.

Bulk isotopic compositions were measured using a Thermo Delta V isotope ratio mass spectrometer coupled to a Costech Elemental Analyser at the Godwin Laboratory for Paleoclimate Research, University of Cambridge. *δ*^13^C and *δ*^15^N values were calibrated relative to VPDB and AIR (respectively) using four internal standards (*Alanine*, *δ*^13^C = − 26.9‰, *δ*^15^N = − 1.4‰; *Nylon*, *δ*^13^C = − 26.3‰, *δ*^15^N = − 1.6‰; *Caffeine*, *δ*^13^C = − 27.5‰, *δ*^15^N =  + 1.0‰; and *BLS*, *δ*^13^C = − 21.6‰, *δ*^15^N =  + 7.3‰) and two international standards (*USGS40*, *δ*^13^C = − 26.39 ± 0.042‰, *δ*^15^N = − 4.5 ± 0.1‰; *IAEA-NO3*, *δ*^15^N =  + 4.7 ± 0.2‰) interspersed through the analytical runs at the following intervals: three aliquots of two types of standards bracketing every 18 samples. Measurement precision was monitored using repeated measurements of all calibration standards and triplicate measurements of all samples. Using the procedure outlined in Szpak et al.^76^, the variability in the calibration standards (s_srm_) was determined to be ± 0.25‰ for *δ*^13^C and ± 0.34‰ for *δ*^15^N and the variability in the replicate measurements (s_rep_) was determined to be ± 0.19‰ for *δ*^13^C and ± 0.05‰ for *δ*^15^N. The overall measure of precision (*u(Rw)*) was calculated to be ± 0.29‰ for *δ*^13^C and ± 0.34‰ for *δ*^15^N.

## Supplementary Information

Supplementary Table S1.

Supplementary Figures.

## Data Availability

All new data discussed in this paper are presented in the “Results” and Supplementary Materials. Data that have been published previously (Liu et al.^19,23^) are acknowledged in the “Materials and methods” and in the Supplementary Materials.
