# Publisher Correction: Localized management of non-indigenous animal domesticates in Northwestern China during the Bronze Age

**DOI:** 10.1038/s41598-021-96994-1

**Published:** 2021-08-26

**Authors:** Petra Vaiglova, Rachel E. B. Reid, Emma Lightfoot, Suzanne E. Pilaar Birch, Hui Wang, Guoke Chen, Shuicheng Li, Martin Jones, Xinyi Liu

**Affiliations:** 1 Department of Anthropology, Washington University in St. Louis, 1 Brookings Dr., St. Louis, MO 63130 USA; 2 Department of Geosciences, Virginia Polytechnic Institute and State University, 926 West Campus Dr., Blacksburg, VA 24061 USA; 3 McDonald Institute for Archaeological Research, University of Cambridge, Downing St., Cambridge, CB2 3ER UK; 4 Department of Anthropology, Department of Geography, University of Georgia, 355 South Jackson Street, Athens, GA 30602 USA; 5 Department of Cultural Heritage and Museology, Fudan University, 220 Handan Rd., Shanghai, 200433 China; 6 Gansu Institute of Cultural Relics and Archaeology, 165 Heping Rd., Lanzhou, 730000 China; 7 Department of Archaeology, Sichuan University, 24 Yihuan Rd. South, Chengdu, 610065 China

Correction to: *Scientific Reports* 10.1038/s41598-021-95233-x, published online 3 August 2021

The original version of this Article contained an error in Figure 1 where panels (a) and (b) were incorrectly captured. The original Figure 1 and accompanying legend appear below.

**Figure 1:**
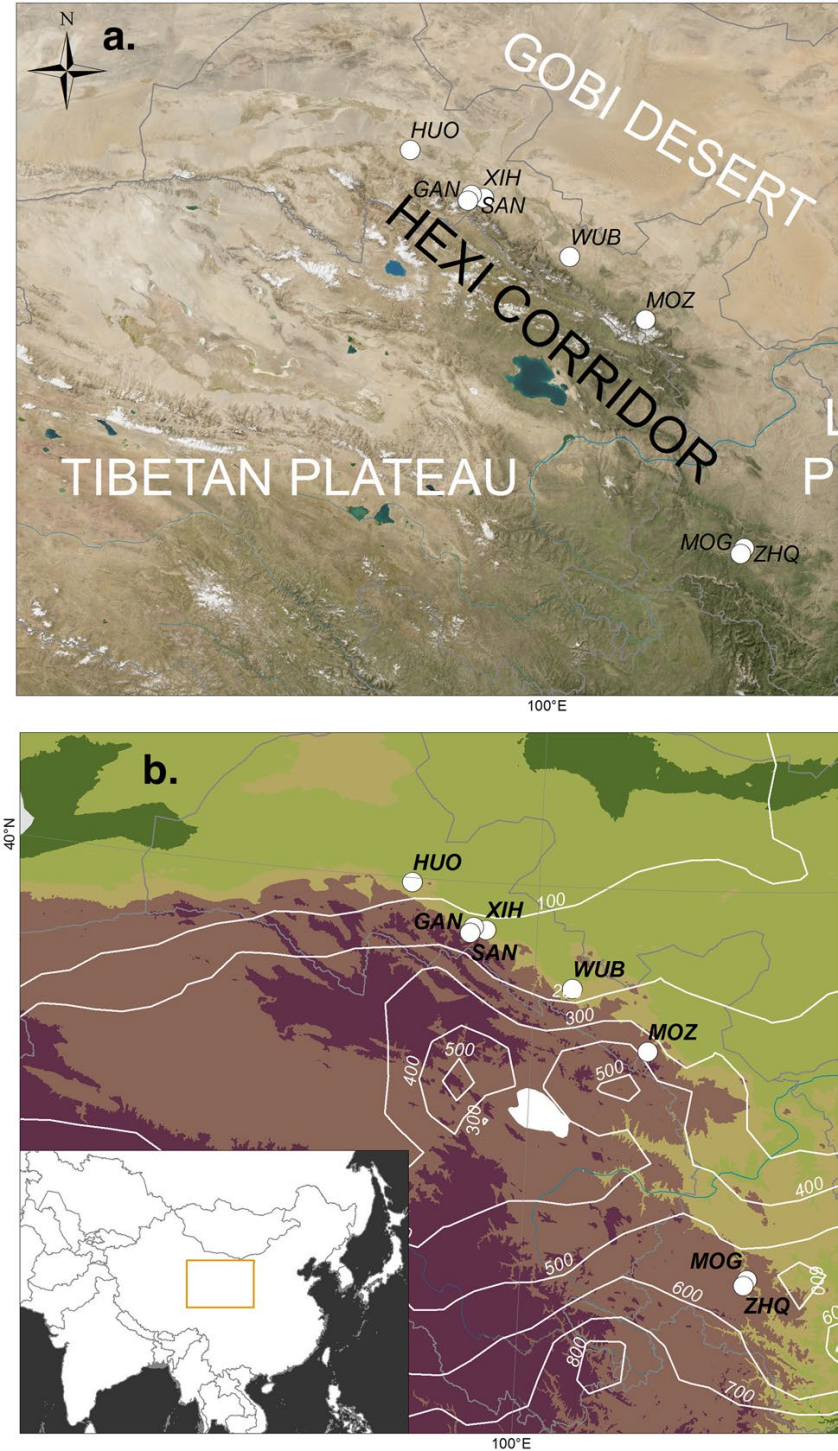
Maps of the study region. (**a**) Topography of the region. (**b**) Precipitation zones (indicated with white contours). *HUO* Huoshaogou, *SAN* Sanbadongzhi, *GAN* Ganguya, *XIH* Xihetan, *WUB* Wuba, *MOZ* Mozuizi, *MOG* Mogou, *ZHQ* Zhanqi. Maps generated using ArcGIS ArcMap 10.2 (https://www.esri.com/about/newsroom/arcwatch/the-best-of-arcgis-10-2/) and public domain data obtained from NASA Blue Marble (https://visibleearth.nasa.gov/collection/1484/blue-marble).

The original Article has been corrected.

