# Supplementary material for: Localized management of non-indigenous animal domesticates in Northwestern China during the Bronze Age
Source: Sci Rep. 2021 Aug 3;11:15764. doi: 10.1038/s41598-021-95233-x (PMC8333310; doi:10.1038/s41598-021-95233-x)
Supplement: Supplementary file 2 — Supplementary Figures. [file 41598_2021_95233_MOESM2_ESM.pdf]

Localized management of non-indigenous animal domesticates in Northwestern China during the Bronze Age

Authors
Petra Vaiglova[1*], Rachel E. B. Reid[1,2], Emma Lightfoot[3], Suzanne E. Pilaar Birch[4], Hui Wang[5], Guoke Chen[6], Shuicheng Li[7], Martin Jones[3], Xinyi Liu[1*]

SUPPLEMENTARY FIGURES

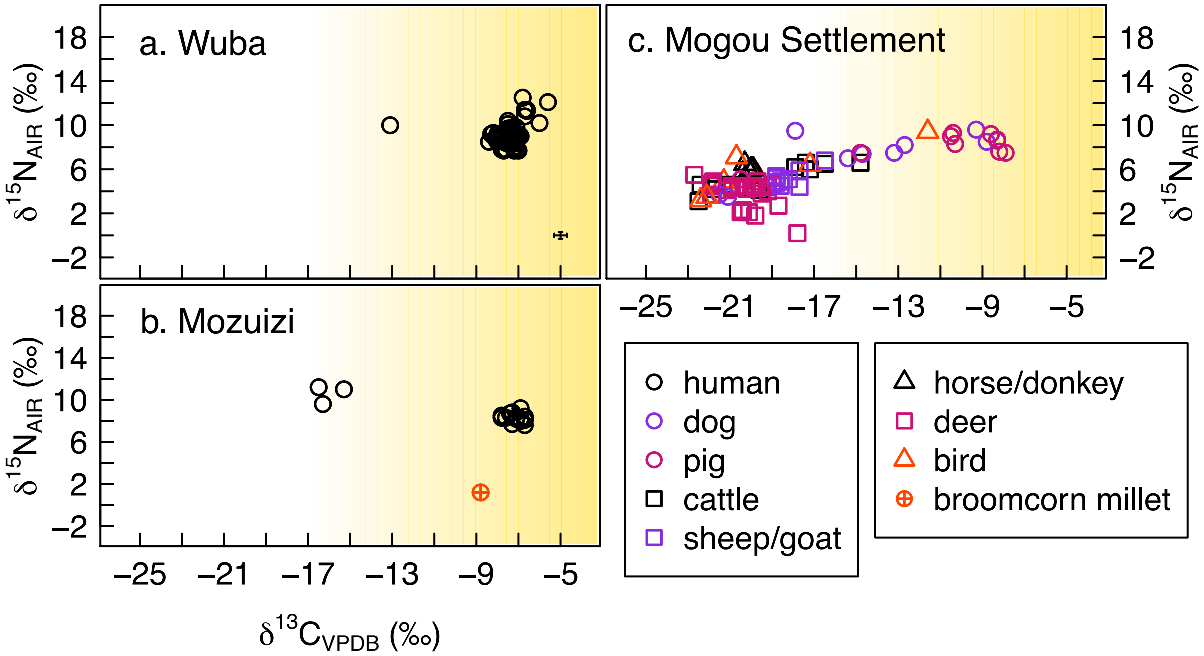

Fig.S1 Stable isotope (carbon, nitrogen) results from sites pre-dating 1900 cal BCE. Bivariate plots of all human, plant, and animal $\delta^{13}C$ and $\delta^{15}N$ values from **(a.)** Wuba, **(b.)** Mozuizi, and **(c.)** Mogou Settlement. The shading indicates increasing input of C_4 vegetation in consumer tissues, with the cut-off set to -17 ‰. Measurement error shown in the bottom-right corner of panel a. See Table 1 for a breakdown of sample numbers.

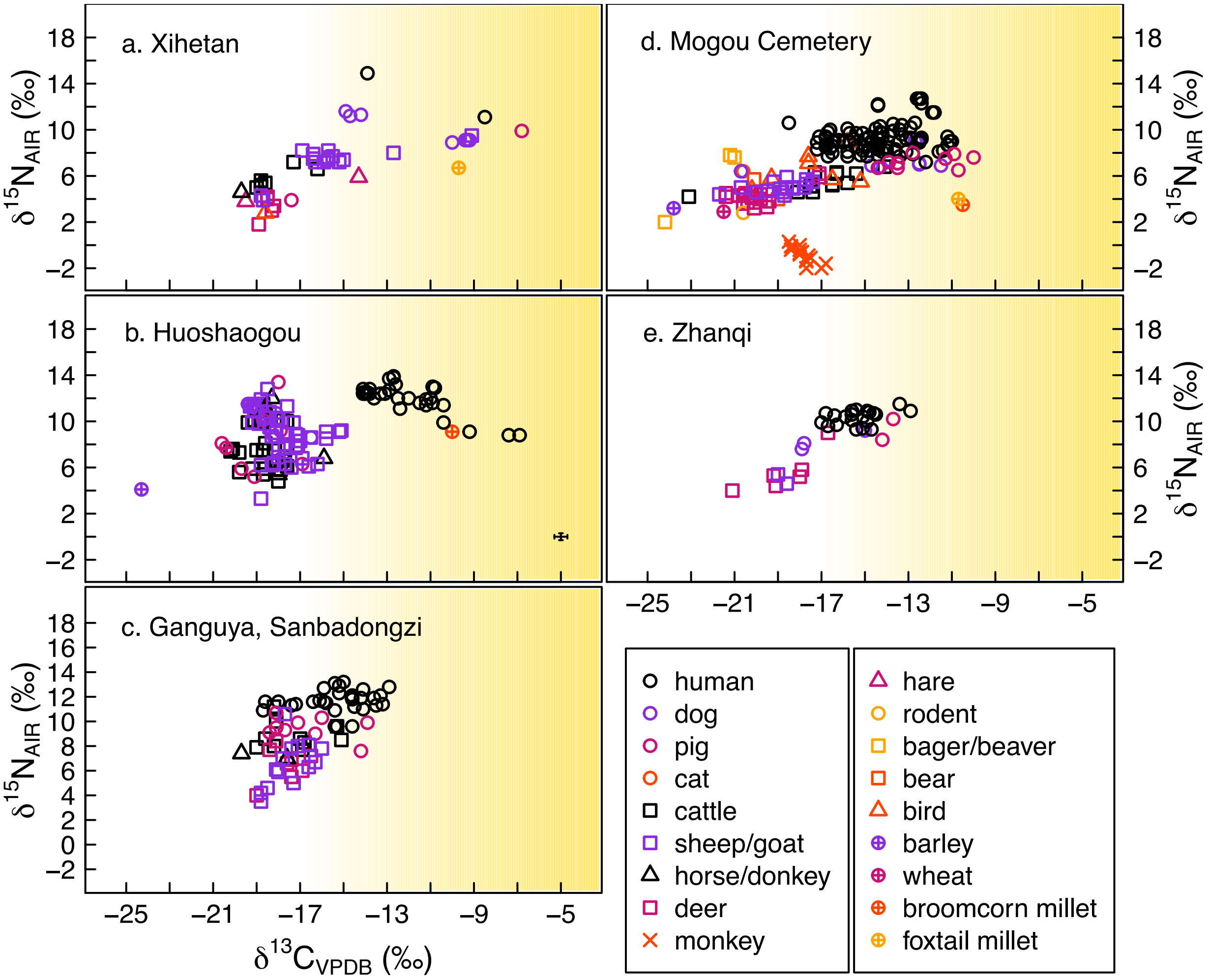

Fig.S2 Stable isotope (carbon, nitrogen) results from sites post-dating 1900 cal BCE. Bivariate plots of all human, plant, and animal $\delta^{13}C$ and $\delta^{15}N$ values from **(a.)** Xihetan, **(b.)** Huoshaogou, **(c.)** Ganguai and Sanbadongzi, **(d.)** Mogou Cemetery, and **(e.)** Zhanqi. The shading indicates increasing input of C_4 vegetation in consumer tissues, with the cut-off set to -17 ‰. Measurement error shown in the bottom-right corner of panel b. See Table 1 for a breakdown of sample numbers.
